# mTOR Inhibition Leads to Src-Mediated EGFR Internalisation and Degradation in Glioma Cells

**DOI:** 10.3390/cancers12082266

**Published:** 2020-08-13

**Authors:** Barbara Colella, Mayra Colardo, Gianna Iannone, Claudia Contadini, Cristina Saiz-Ladera, Claudia Fuoco, Daniela Barilà, Guillermo Velasco, Marco Segatto, Sabrina Di Bartolomeo

**Affiliations:** 1 Department of Biosciences and Territory, University of Molise, 86090 Pesche (IS), Italy; b.colella@studenti.unimol.it (B.C.); m.colardo@studenti.unimol.it (M.C.); g.iannone@studenti.unimol.it (G.I.); marco.segatto@unimol.it (M.S.); 2 Department of Biology, University of RomeTor Vergata, 00133 Rome, Italy; claudiacontadini@gmail.com (C.C.); claudia.fuoco@uniroma2.it (C.F.); daniela.barila@uniroma2.it (D.B.); 3 Laboratory of Cell Signaling, Istituto di Ricovero e Cura a carattere Scientifico (IRCSS) Fondazione Santa Lucia, 00179 Rome, Italy; 4 Department of Biochemistry and Molecular Biology, School of Biology, Complutense University and Instituto de Investigaciones Sanitarias San Carlos (IdISSC), 28040 Madrid, Spain; crsaiz04@gmail.com (C.S.-L.); gvelasco@quim.ucm.es (G.V.)

**Keywords:** mTOR, EGFR, glioma, autophagy

## Abstract

Epidermal Growth Factor receptor (EGFR) is a tyrosine kinase receptor widely expressed on the surface of numerous cell types, which activates several downstream signalling pathways involved in cell proliferation, migration and survival. EGFR alterations, such as overexpression or mutations, have been frequently observed in several cancers, including glioblastoma (GBM), and are associated to uncontrolled cell proliferation. Here we show that the inhibition of mammalian target of Rapamycin (mTOR) mediates EGFR delivery to lysosomes for degradation in GBM cells, independently of autophagy activation. Coherently with EGFR internalisation and degradation, mTOR blockade negatively affects the mitogen activated protein/extracellular signal-regulated kinase (MAPK)/ERK pathway. Furthermore, we provide evidence that Src kinase activation is required for EGFR internaliation upon mTOR inhibition. Our results further support the hypothesis that mTOR targeting may represent an effective therapeutic strategy in GBM management, as its inhibition results in EGFR degradation and in proliferative signal alteration.

## 1. Introduction

Epidermal Growth Factor receptor (EGFR), also known as ErbB1 or HER1, belongs to the tyrosine kinase receptors family (RTK) and regulates epithelial tissue development and homeostasis [1]. In physiological conditions, inactive RTK receptors continuously travel through the endocytic compartment, by slow internalisation and recycling to the plasma membrane. EGF binding stimulates EGFR activation and signal transduction inside the cell, thus regulating cell growth, differentiation, proliferation and migration [2,3]. In particular, upon ligand engagement, activated receptors are subjected to clathrin-mediated (CME) or clathrin-independent (CIE) endocytosis depending on ligand abundance, and spend most of their remaining lifetime travelling the vesicular network of endosomes [4,5,6,7,8]. Once internalised in early endosomes, EGFR can be recycled to the cell surface or delivered to lysosomes for degradation, in a delicate balance between continuous signalling from cell surface/endosomal compartments and signal attenuation from the degradative route [4,9].

It has been demonstrated that activated RTK still retains the ability to continuously signal within the vesicular compartments until the receptors are recycled back to the cell surface or taken up into lysosomes for degradation [9].

EGFR alterations have been reported in several diseases, particularly, in cancer [10]. Indeed, EGFR gene is frequently mutated, amplified or overexpressed in various kinds of tumours including Glioblastoma multiforme (GBM), the most aggressive brain tumour in adults [10,11]. In particular, in GBM, EGFR is amplified in 40%, overexpressed in 60% and mutated or deleted in 24–67% of primary tumours [12]. Deletion of exon 2–7, also known as variant III (EGFR vIII), represents the most frequent mutation occurring in GBMs, found in up to 45% of cases [13,14]. Mutated EGFR are usually constitutively active, ligand-independent receptors with altered trafficking and down-regulation, resulting in aberrant downstream signal transduction which promotes tumour development [15,16]. Receptor overexpression, which is often associated to an enhanced expression of cognate ligands [17], is also associated to receptor turnover alterations and to increased downstream signalling [18,19].

Mammalian target of Rapamycin (mTOR) kinase is part of two multi-protein complexes, known as mTORC1 and mTORC2, that act as nutrients sensors in the cell, regulating protein synthesis through a phosphorylation cascade [20]. Aberrant mTOR activation is linked to tumour proliferation and invasion. For this reason, mTOR is recurrently proposed as a putative pharmacological target, and several specific inhibitors, mainly directed to mTORC1, have been developed and tested in the last decade [21]. mTOR also regulates autophagy, a lysosome-degradation system crucial to the preservation of cellular homeostasis [22]. As a nutrient sensor, mTOR is normally activated under fed conditions, leading to autophagy inhibition. Conversely, nutrient starvation results in mTOR inactivation and in a subsequent autophagy induction. In our previous work, we demonstrated that autophagy induction by nutrient starvation or by mTOR inhibition impairs migration and invasion in glioblastoma cells through reversing the Epithelial-to-Mesenchymal Transition (EMT) process and inhibiting the Wnt/β-catenin pathway [23,24,25].

It is now well established that RTK signalling regulates PI3K/mTOR pathway and autophagy [26]. However, little evidence indicates the possible existence of an opposite relation [27,28]. In this work, we evaluated the prospective modulation of mTOR on EGFR activity and turnover. We found that mTOR inhibition delocalises EGFR from the plasma membrane to the cytoplasm in GBM cellular models. We also observed that the receptor is delivered to lysosomes for degradation, independently of autophagy. These events were also accompanied by the suppression of the mitogen activated protein/extracellular signal-regulated kinase (MAPK)/ERK pathway, suggesting that mTOR inhibition may affect EGFR ability to endorse GBM cell survival and proliferation.

## 2. Results

### 2.1. mTOR Inhibition Induces EGFR Relocalisation in GBM Cells

**Figure 1:**
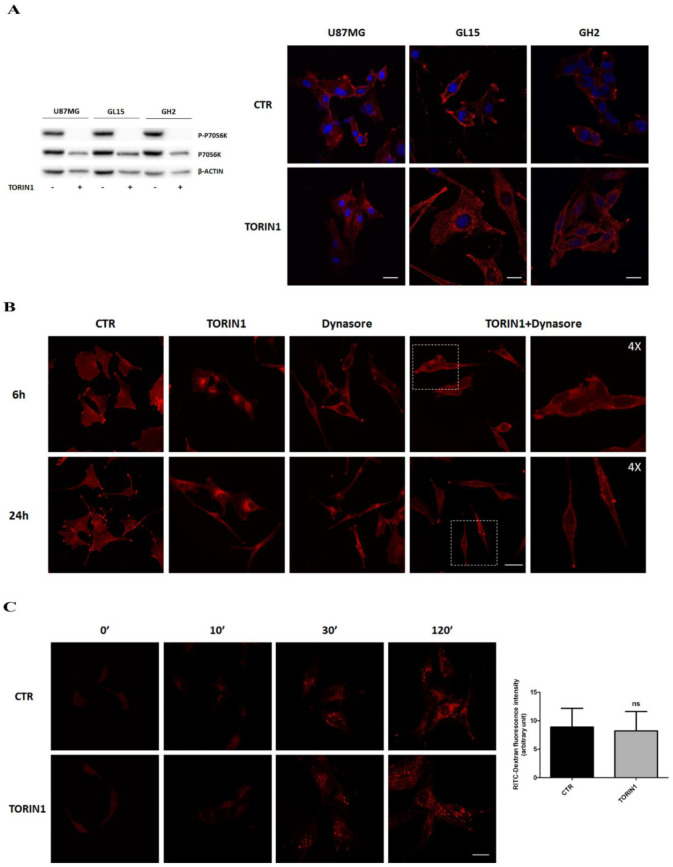
Epidermal Growth Factor receptor (EGFR) internalises into Glioblastoma multiforme (GBM) cells upon mTOR inhibition. (**A**) U87MG (upper panels), GL15 (middle panels) and primary GH2 cells (lower panels) were cultured in complete medium (DMEM) (CTR) or in DMEM in the presence of 250 nM Torin1 for 18 h. Immunocytochemistry and confocal analysis for EGFR localisation (red) were then performed. Hoechst 33342 was used to stain nuclei (blue). Scale bar, 30 μM. Western blot analysis of P-p70S6K and p70S6K was performed to check mTOR pathway inhibition by Torin1. β-actin was used as loading control. The blots are representative of three independent experiments. (**B**) Immunocytochemistry and confocal analysis for EGFR localisation (red) were performed in U87MG cells, upon 6 h and 24 h Torin1 treatment in the presence or absence of 100 μM Dynasore. Scale bar, 30 μM. A 4× magnification is shown for the right panels representing cells treated with Torin1 plus Dynasore. (**C**) U87MG cells were incubated with 0.5 mg/mL Rhodamine Dextran for the indicated times and its uptake within the cells analysed by image capturing at the confocal microscope. Scale bar, 30 μM. Fluorescence quantification of Dextran uptake at 120′ is shown in the right panel.

Similarly to other tumours, GBM is characterized by an aberrant EGFR expression and activation, leading to de-regulated downstream signalling pathways. In order to investigate a putative impact of autophagic stimuli on EGFR expression and signalling in GBM, we cultured U87MG and GL15 GBM cells in the presence of Torin1 and AZD8055, two inhibitors of both mTORC1 and mTORC2 complexes, or in EBSS (amino acid- and serum- free medium) for 18 h. GH2 primary cells were also cultured in complete medium or in the presence of Torin1, for 18 h. As shown in Figure 1A and Figure S1A (left panels), mTOR inhibition obtained by cell exposure to all the stimuli was confirmed by the strong decrease in the phosphorylated forms of its substrate P70S6K, observed by Western blotting analysis. Next, we checked for EGFR subcellular localisation by immunocytochemistry and we found, as expected, that the receptor was mainly localized at plasma membrane of control cells; conversely, cells grown in the presence of autophagy stimuli did not show EGFR membrane exposure (Figure 1A, right panels, and Figure S1A). Chiefly, an EGFR punctate immunostaining pattern was observed in discrete areas within the cytoplasm of Torin1 and AZD8055-treated cells (Figure 1A and Figure S1A). It is noteworthy the receptor signal was strongly reduced after 18 h of EBSS treatment and some peri-nuclear foci were detected likely due to the modulation of multiple signalling pathways upon FBS and amino acid deprivation in this condition [29] (Figure S1A). A time-course analysis revealed that EGFR re-localisation was already evident upon 2 and 4 h mTOR inhibition with Torin1 (Figure S1B). In order to investigate the mechanism of EGFR relocalisation, glioma cells were pre-incubated with the endocytosis inhibitor Dynasore before Torin1 stimulation and analysed after 6 and 24 h. We observed that, upon Dynasore administration, the receptor internalisation was largely prevented, demonstrating that mTOR inactivation induces a burst of EGFR endocytosis (Figure 1B and Figure S1C). Experiments performed with fluorescent Dextran (70 kDa size) suggest that the clathrin- and dynamin-independent endocytic flux is not altered upon mTOR inhibition, and that the clathrin- and dynamin-dependent endocytosis is involved in EGFR internalisation (Figure 1C). Taken together the results collected in Figure 1 and in Figure S1 suggest that mTOR inhibition stimulates EGFR endocytosis likely via clathrin-and dynamin-dependent mechanisms.

### 2.2. EGFR De-Localisation Is Not Dependent on Canonical Autophagy in GBM Cells

**Figure 2:**
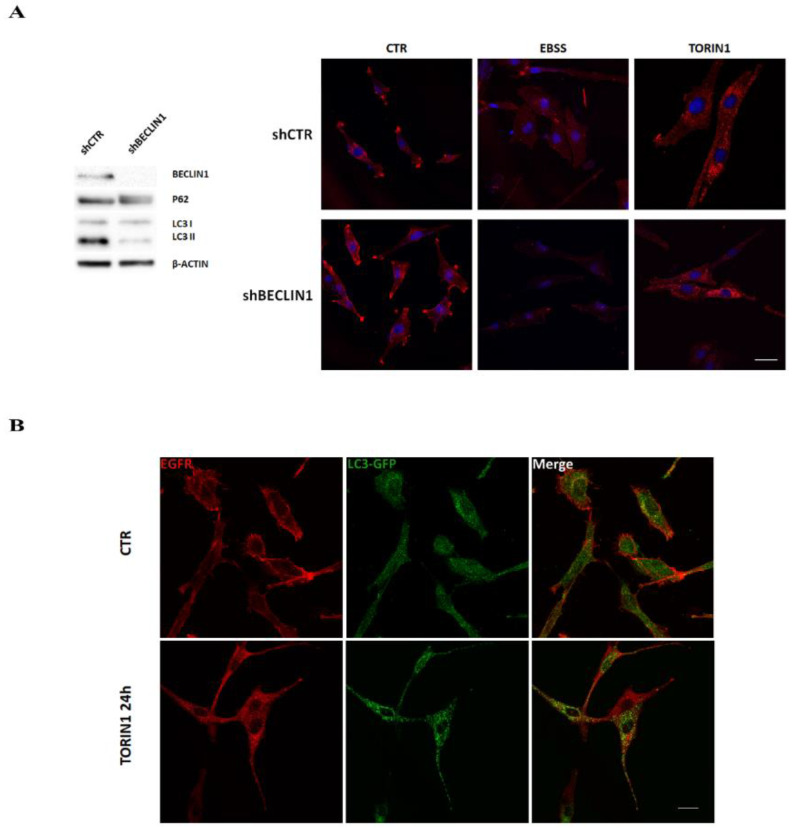
EGFR de-localisation in GBM cells is independent of canonical autophagy. (**A**) shCTR and shBECLIN1 GL15 cells [23] were cultured in DMEM (CTR) or amino acid- and serum- free medium (EBSS) media or in DMEM in the presence of 250 nM Torin1 for 18 h and subjected to immunocytochemistry and confocal analysis for EGFR localisation (red) (right panels). Hoechst 33342 was used to stain nuclei (blue). Scale bar, 30 μM. Western blot analysis of P62 and LC3 I/II was also performed in basal conditions to check autophagy status (left panel). A specific antibody for BECLIN1 was used to check the silencing efficiency. β-ACTIN was used as loading control. The blot is representative of three independent experiments. (**B**) U87MG cells were transduced with GFP-LC3-expressing retrovirus as described in Material and Methods. Infected cells, cultured in DMEM alone (CTR) or in DMEM containing 250 nM Torin1 for 24 h, were subjected to immunocytochemistry and confocal analysis for EGFR (red) and autophagosomes (green) localisation. Colocalisation was excluded by calculating the Pearson’s correlation coefficient r (mean r CTR, 0.15 ± 0.02; Torin1, 0.2 ± 0.03). The images showing the merge of the two signals are shown in the right panels. Scale bar, 30 μM.

In order to discriminate between an autophagy-dependent or -independent effect of mTOR inhibitors on EGFR de-localisation, a Beclin1-silenced GL15 cell line was employed [23]. In comparison to control cells, GL15 shBeclin1 are less able to accumulate LC3II (the autophagosome-associated form of LC3) and to degrade the autophagy substrate P62 [30], in both basal (Figure 2A, left panel) and starved conditions [23], in line with the role of Beclin1 for canonical autophagy [24]. Interestingly, Beclin1 silencing did not prevent EGFR de-localisation upon EBSS incubation or Torin1 treatment for 18 h (Figure 2A, right panels). To confirm an autophagy-independent role of mTOR on EGFR trafficking, we infected U87MG cells with a GFP-LC3 expressing retrovirus and analysed EGFR localisation upon autophagy stimulation. Immunofluorescence shows the appearance of the typical GFP-LC3 dots, identifying autophagosomes, 24 h after Torin1 treatment (Figure 2B). Notably, GFP-LC3 puncta did not co-localize with EGFR intracellular signal in Torin1-treated cells, thus excluding a re-localisation of the receptor within autophagosomes (Figure 2B). These results indicate that mTOR activity is involved in EGFR internalisation and trafficking, independently of its role in autophagy regulation, and that EGFR does not re-localise to autophagosomes upon mTOR inhibition.

### 2.3. mTOR Inhibition Targets EGFR to Lysosomes

Next, we investigated the subcellular compartment involved in EGFR translocation upon mTOR inhibition. To this aim, we performed immunofluorescence analyses by using specific subcellular markers for cytoplasmic organelles, that are known to be involved in RTK trafficking [4].

**Figure 3:**
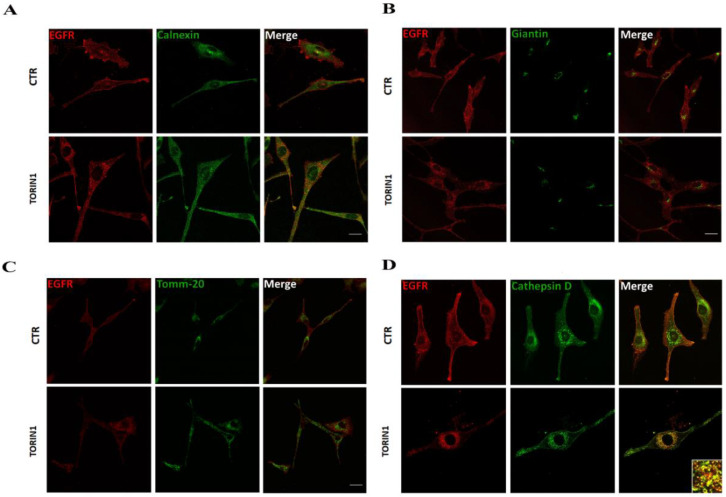
EGFR is delivered to lysosomes upon mTOR inhibition in GBM cells. U87MG treated with Torin1 for 24h or untreated (CTR) were subjected to immunocytochemistry and confocal analysis for EGFR (red) and for different subcellular markers (green): Calnexin for endoplasmic reticulum (**A**), Giantin for Golgi cisternae (**B**), Tomm-20 for mitochondria (**C**) and Cathepsin D for lysosomes (**D**). The images showing the merge of the two signals are shown in the right panels. Inset containing a high magnification view of the merge image is also shown. Scale bar, 30 μM. Colocalisation was assessed by calculating the Pearson’s correlation coefficient r (mean r in D: CTR, 0.2 ± 0.03; Torin1, 0.75 ± 0.06).

We observed that EGFR signal is largely separated from that of the endoplasmic reticulum, Golgi cisternae and mitochondria upon mTOR inhibition in U87 cells, as shown in Figure 3A–C. By contrast, a great amount of EGFR was detected in correspondence to CathepsinD-positive puncta, indicating a lysosomal delivery of the receptor 24 h after Torin1 treatment (Figure 3D). The occurrence of EGFR localisation to lysosomes upon Torin1 administration was also observed in GL15 cells and in primary GH2 cells (Figure S2). This figure indicates that, among the subcellular compartments analysed, EGFR relocalises to lysosomes upon mTOR inhibition.

### 2.4. EGFR Expression Is Reduced upon mTOR Inhibition in GBM Cells

**Figure 4:**
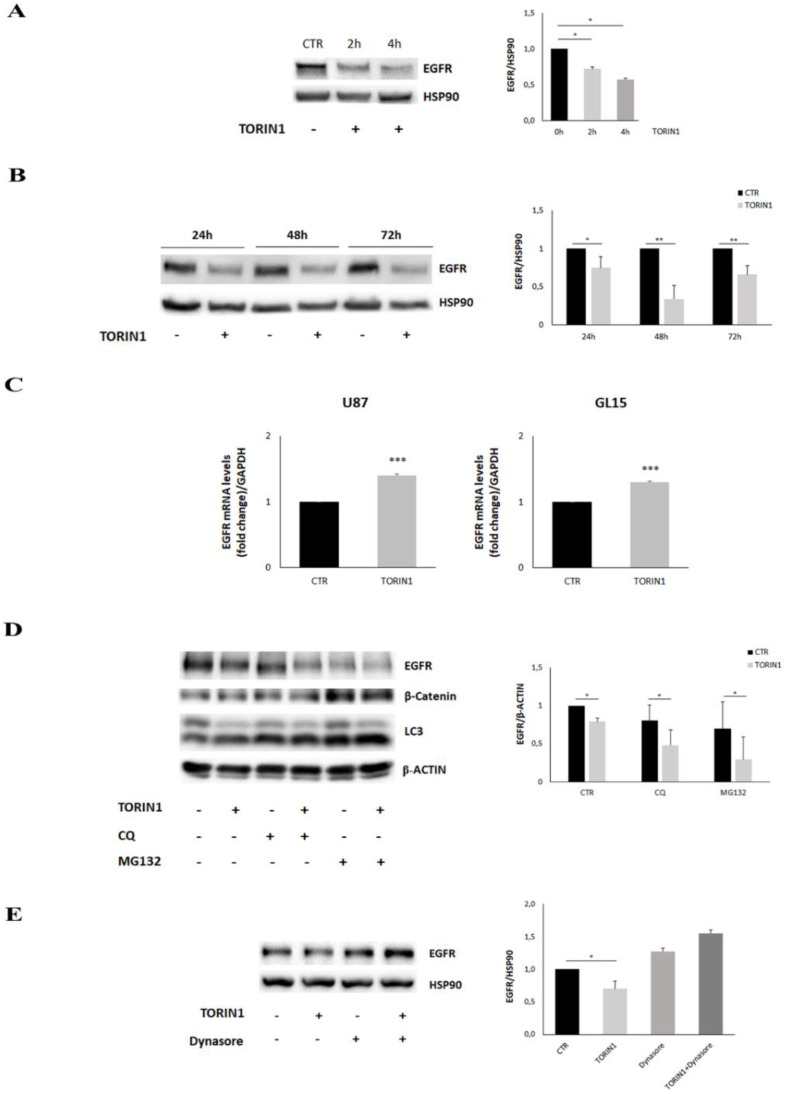
mTOR inhibition leads to a reduction of EGFR expression in GBM cells. (**A**,**B**) U87MG cells were cultured in complete DMEM medium in the presence (TORIN1) or absence (CTR) of 250 nM Torin1 and analysed at the indicated time points. Western blot analyses were performed by using a specific antibody for EGFR. HSP90 was used as loading control. (**C**) Real time experiments were performed on U87MG and GL15 cells stimulated or not with 250 nM Torin1 for 24 h. GAPDH was used as internal control. The graph represents the mean ± SD of three different experiments: *** *p* < 0.001 Student *t*-test. (**D**) U87MG cells were stimulated (+) or not (−) with 250 nM Torin1 for 24 h in the presence of 20 μM Chloroquine (CQ) or 3 μM MG132 and Western blot analyses were performed by using antibodies for EGFR, β-catenin, LC3 and β-actin. (**E**) U87MG cells were stimulated (+) or not (−) with 250 nM Torin1 for 24 h in the presence of 100 μM Dynasore and Western blot analysis was performed by using antibodies for EGFR and HSP90 was used as loading control. The graphs represent the mean ± SD of three different experiments. Statistical significance: * *p* < 0.05 Student *t*-test ** *p* < 0.01 Student *t*-test.

The obtained results prompted us to verify whether the mTOR inhibition could produce any effects on EGFR protein expression. Thus, we analysed the expression level of the receptor in U87MG and GL15 cells cultured in the presence or absence of Torin1. Western blotting analysis indicated a significant reduction of EGFR expression in 2 and 4 h after Torin1 exposure (Figure 4A and Figure S3A) which persisted up to 72 h (Figure 4B and Figure S3B), compared to control cells. Likewise, the reduction of EGFR protein levels was also observed in primary GH2 cells treated with Torin1 for 2, 4 and 24 h (Figure S3C). These results suggest that the mTOR inhibition induces EGFR degradation, in line with the observation of EGFR lysosomal delivery. We also checked for a possible effect of Torin1 on EGFR transcription and 24 h after drug administration, we observed a mild but significant mRNA increase in the cell lines analysed (Figure 4C). The build-up in the EGFR transcript suggests a possible compensatory mechanism to counteract the reduction in protein levels and could explain what causes the endency of EGFR protein to partially increase at late time points. In order to investigate the mechanism of EGFR degradation, we performed Torin1 treatments in the presence of MG132, Chloroquine and dynasore to inhibit, respectively, proteasome, autophagy and endocytosis. Figure 4D shows that both autophagy and proteasome inhibition did not induce any change in Torin1-stimulated cells, whereas endocytosis inhibition fully prevented EGFR decrease, as expected (Figure 4E). Peculiarly, we observed a high variability of EGFR expression in Chloroquine (CQ)- and particularly in MG132-treated cells, with a non-significant tendency to decrease, maybe due to the toxicity of the drugs (Figure 4E). We also observed that mTOR inhibition did not induce an accumulation of ubiquitinated EGFR, as differently observed for other proteins [31] (Figure S3D). Taken together, the results illustrated indicate that mTOR inhibition stimulates EGFR degradation and its novel transcription, independently of proteasome and autophagy activation.

### 2.5. mTOR Inhibition Leads to MAPK/ERK Pathway Down-Regulation in GBM Cells

**Figure 5:**
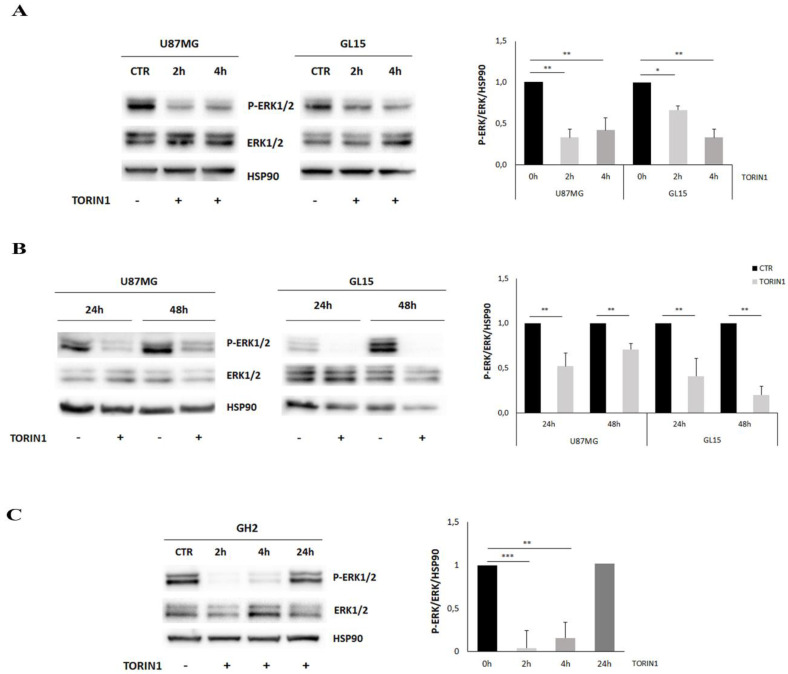
The mitogen activated protein (MAPK)/ERK pathway is down-regulated upon mTOR inhibition in GBM cells. (**A**,**B**) U87MG, GL15 were cultured in complete DMEM medium in the presence (TORIN1) or absence (CTR) of 250 nM Torin1 and analysed at 2 h and 4 h (**A**) and at 24 h and 48 h (**B**). Western blot analysis was performed by using specific antibodies for ERK1/2 and P-ERK1/2. HSP90 was used as loading control. (**C**) GH2 cells were cultured in complete DMEM medium in the presence (TORIN1) or absence (CTR) of 250 nM Torin1 and analysed at the indicated time points. Western blot analysis was performed by using specific antibodies for ERK1/2 and P-ERK1/2. HSP90 was used as loading control. The graph represents the mean ± SD of three different experiments. Statistical significance: * *p* < 0.05 Student *t*-test ** *p* < 0.01 Student *t*-test, *** *p* < 0.001 Student *t*-test.

EGFR is involved in cell proliferation, growth and differentiation, through the activation of several downstream signal transduction pathways, including the MAPK/ERK cascade. Therefore, we checked whether the EGFR de-localisation and degradation occurring upon mTOR inhibition also affected ERK activity. To address this aim, U87MG and GL15 were grown in the presence or absence of Torin1 at different time points, and Western blotting analysis was performed, by taking advantages of antibodies recognizing the phosphorylated, activated, forms of ERK1/2. As shown in Figure 5A, ERK1/2 phosphorylation was strongly and persistently suppressed in Torin1-treated cells, being observable at 2 and 4 h after treatment, and maintained up to 48 h in U87MG and in GL15 (Figure 5B). Moreover, the dependence of ERK modulation on mTOR inhibition was further evaluated in GBM primary cells. Similarly to U87MG and GL15 cell lines, ERK1/2 activation was drastically reduced in GH2 cells after 2 and 4 h following Torin1 treatment, although it increased again after 24 h exposure (Figure 5C). In summary, the MAP/ERK pathway is strongly attenuated upon mTOR inhibition in our GBM models.

### 2.6. Src Activity Is Required to EGFR Internalisation in GBM Cells

**Figure 6:**
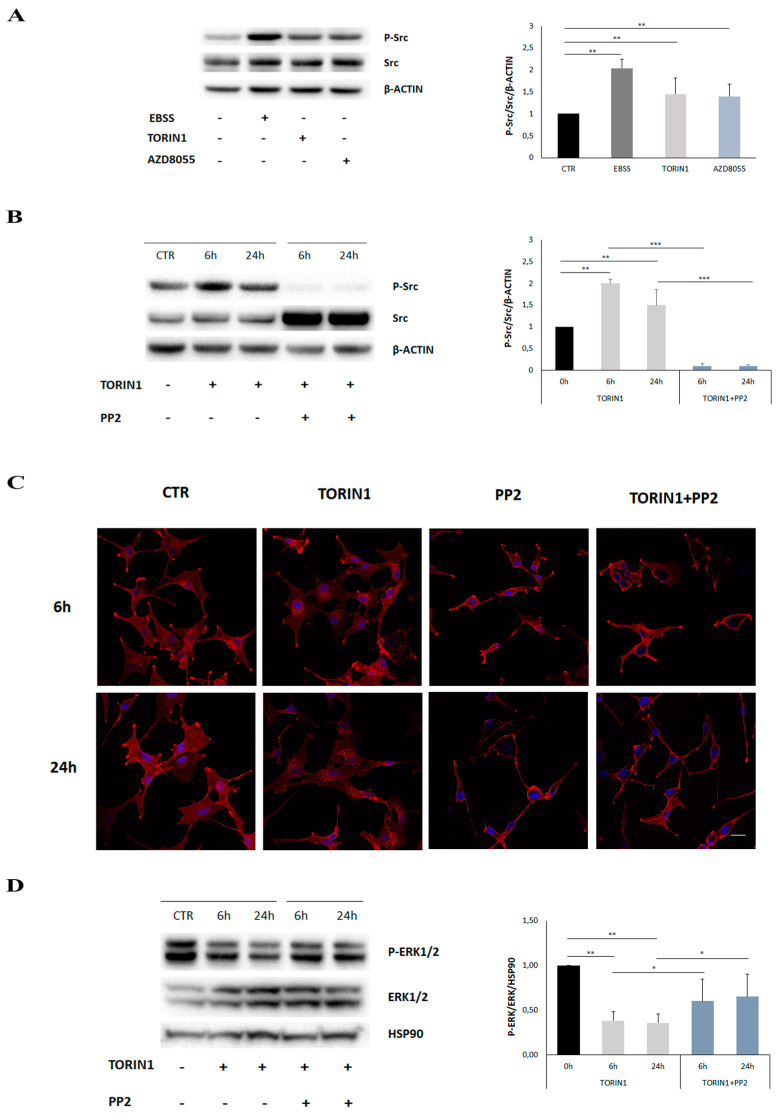
mTOR inhibition leads to SRC activation. (**A**) U87MG cells were cultured in complete DMEM (CTR) or aminoacid- and serum- free medium (EBSS) or in DMEM in the presence of 250 nM Torin1 or 100 nM AZD8055 for 18 h. Western blot analysis was performed by using specific antibodies for the total (SRC) and the phosphorylated (Y416) form of SRC (P-Src). β-ACTIN was used as loading control. (**B**) U87MG cells were cultured in DMEM in the presence of Torin1 pre-incubating or not with 20 µM PP2 inhibitor for the indicated time points. Western blot analysis was performed by using specific antibodies for the total (SRC) and the phosphorylated (Y416) form of SRC (P-Src). β-ACTIN was used as loading control. The graphs represent the mean ± SD of three different experiments. Statistical significance: ** *p* < 0.01 Student *t*-test, *** *p* ≤ 0.001 Student *t*-test (**C**) Immunocytochemistry and confocal analysis for EGFR localisation (red) were performed in U87MG, upon 6 h and 24 h of Torin1 and PP2 treatments, as indicated. Hoechst 33342 was used to stain nuclei (blue). Scale bar, 30 μM. (**D**) U87MG cells were cultured in DMEM in the presence of Torin1 pre-incubating or not with 20 µM PP2 inhibitor for the indicated time points. Western blot analysis was performed by using specific antibodies for the total (ERK1/2) and the phosphorylated form of ERK1/2 (P-ERK1/2). HSP90 was used as loading control. The graph represents the mean ± SD of three different experiments. Statistical significance: * *p* < 0.05 Student *t*-test, ** *p* < 0.01 Student *t*-test.

RTK endocytosis is regulated by clathrin-dependent and -independent mechanisms. It has been shown that Src kinase plays a role in regulating EGFR endocytosis by phosphorylating a clathrin heavy chain following EGF binding to its receptor [32,33]. Thus, we checked whether Src activity was involved in EGFR internalisation upon mTOR inhibition. First, we performed Western blotting analysis by using an antibody raised against the phosphorylated and active form of Src on protein extracts of U87MG incubated with EBSS, Torin1 or AZD8055, or not, for 18 h. As shown in Figure 6A, mTOR inhibition resulted in Src activation upon exposure to all the mTOR-inhibitory stimuli employed in this study. The results shown suggest that SRC activity is crucial for EGFR internalisation within the cells and for MAP/ERK pathway inhibition upon mTOR inhibition.

Src activation was already evident after 6 h of Torin1 treatment and was completely prevented by co-administration of the Src inhibitor PP2 (Figure 6B). Notably, EGFR internalisation was completely abrogated when cells were incubated with PP2 at both 6 and 24 h, indicating the involvement of Src activation in mTOR-mediated receptor trafficking (Figure 6C). Furthermore, PP2 pre-incubation partially prevented ERK1/2 dephosphorylation induced by Torin1 at 6 and 24 h (Figure 6D).

### 2.7. mTOR Blockade Inhibits Cell Proliferation and Sensitized GBM Cells to Temozolomide

**Figure 7:**
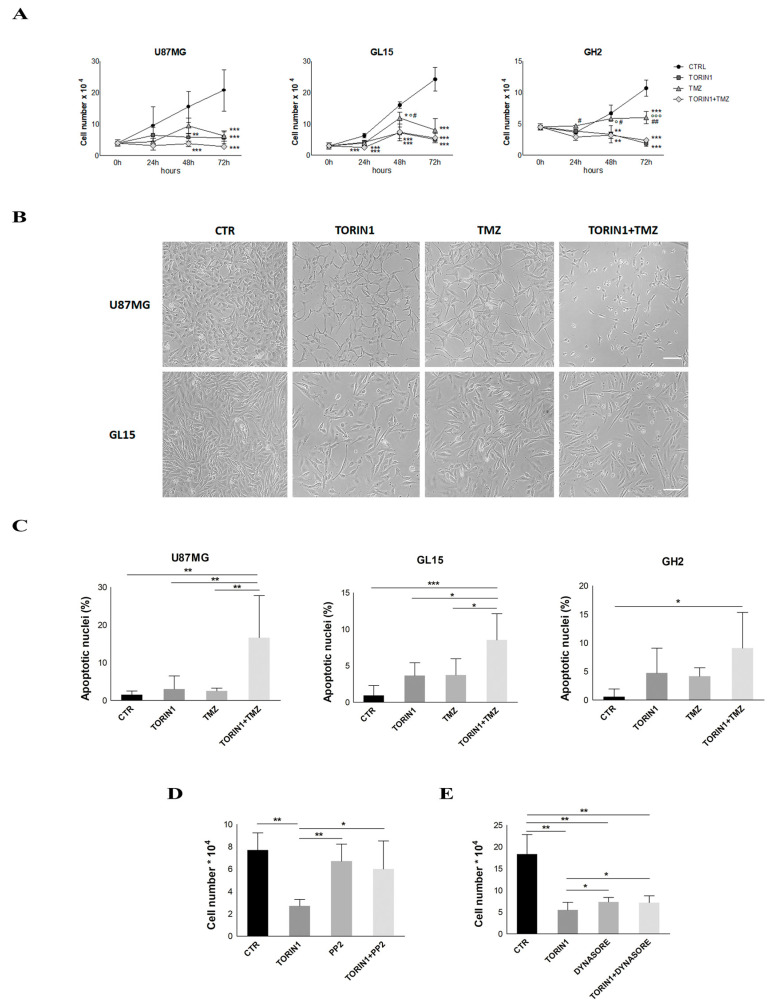
mTOR inhibition leads to cell proliferation arrest. (**A**) U87MG, GL15 and GH2 cells were cultured in complete DMEM (CTR) or in the presence of 250 nM Torin1 (TORIN1) or 500 µM Temozolomide (TMZ) or both. At the indicated time points, cells were trypsinised and counted in a Newbauer chamber. The graph represents the mean ± Standard Error of the Mean (SEM) of three different experiments. Statistical significance: * indicates significance vs. Ctr; # indicates significance vs. Torin1 + TMZ; ° indicates significance vs. Torin1. ** *p* < 0.01 *** *p* ≤ 0.001 Two-way ANOVA. (**B**) Pictures of the cells in (**A**) were taken at 72 h of the indicated treatments. Scale bar: 100 µm (**C**) U87MG, GL15 and GH2 cells were cultured in complete DMEM (CTR) or in the presence of 250 nM Torin1 (TORIN1) or 500 µM TMZ or both Torin1 and TMZ for 72 h. Nuclei were then stained with DAPI and counted under fluorescence microscope (20× objective). 10 fields per each condition were counted. The graphs represent the mean ± SD of three different experiments. * *p* < 0.05, ** *p* < 0.01, *** *p* ≤ 0.001 One-way ANOVA; (**D**,**E**) U87MG cells were incubated with Torin1 in the presence or absence of 20 µM PP2 (**D**) or in the presence of 100 μM Dynasore (**E**) for 48 h before cell counting in a Newbauer chamber. The graphs represent the mean ± SD of three different experiments. Statistical significance: * *p* < 0.05, ** *p* < 0.01 One-way ANOVA.

Next, we investigated whether mTOR inhibition could affect cell proliferation and viability in our GBM models. Indeed, experimental evidence indicates that mTOR inhibitors negatively affect cell proliferation and viability [34,35,36]. Cell counts were performed at 24, 48 and 72 h following Torin1 treatment and we found that cell proliferation was strongly affected starting from 24 h in all the cell lines analysed (Figure 7A,B). We also incubated cells with Temozolomide (TMZ), a drug commonly used in GBM therapy, in the presence or absence of Torin1. As shown in Figure 7A,B, TMZ impaired cell proliferation starting from 48 h of stimulation and Torin1 co-incubation slightly, but not significantly potentiated its anti-proliferative activity (Figure 7A,B). Nuclear staining with 4′,6-diamidino-2-phenylindole (DAPI) revealed that Torin1, per se, did not induce a significant increase in apoptosis up to 72 h of stimulation, similarly to TMZ alone; however, the combination of Torin1 and TMZ resulted in a significative increase in apoptotic nuclei at 72 h (from 1.5 to 16.6% in U87MG; from 0.9 to 8.5% in GL15; from 0.6 to 9.1% in GH2) (Figure 7C). In order to correlate the effects on cell proliferation with EGFR internalisation, we also performed cell counting experiments in Torin1-stimulated cells in the presence of the SRC inhibitor PP2 or of the endocytosis inhibitor Dynasore. We observed that pre-incubation with both PP2 and Dynasore partially prevented Torin1-induced arrest of cell proliferation, although Dynasore negatively impacted, per se, cell proliferation (Figure 7D,E). Taken together, these results indicate that mTOR inhibition negatively affects cell proliferation and this effect is dependent on EGFR internalisation within the cell. Moreover, mTOR inhibition potentiates the ability of TMZ to induce apoptosis.

## 3. Discussion

EGFR is one of the most studied RTK members due to its well-known oncogenic activity [10]. For the same reason, during last decade, it has emerged as a powerful target of multiple cancer therapies, some of which have successfully introduced it in clinical practice for some tumour types. A specific group of EGFR deletions, point mutations and amplification, are frequently found in GBM [37]. Specifically, overexpression of this receptor on the cell surface is found in 60% of primary GBM and is associated with the most aggressive GBM phenotypes [12]. Overexpression and oncogenic mutations of EGFR lead to spontaneous dimerization and activation of the receptor, independently of the presence of the ligand [38]. In addition, beside abnormal expression and activation, dysregulated EGFR intracellular trafficking also plays a crucial role in GBM oncogenesis [9].

For the first time, we demonstrated that the inhibition of the mTOR complexes by drug administration or by amino acid depletion strongly induces EGFR disappearance from the plasma membrane and stimulates its endocytosis within GBM cells. The observation that clathrin-independent endocytosis is not altered by mTOR inhibition could suggest a role for mTOR in regulating the clathrin-dependent endocytosis. Receptor delocalisation was still observed in cells defective for the autophagy master gene *BECLIN1*, thus indicating that autophagy induction is not responsible for EGFR de-localisation.

Besides the well-characterized recycling and degradative trafficking pathways, emerging evidence underlies alternative routes for EGFR endocytosis, including traffic to the nucleus, mitochondria and other subcellular compartments [39,40]. Thus, we investigated the fate of the internalized receptor after mTOR inhibition. We did not find any receptor co-localisation with the nucleus, mitochondria, ER and Golgi, whereas a strong overlapping signal was observed into lysosomes. EGFR positivity was not observed into autophagosomes, thus confirming that receptor delivery to the degradative compartment is not mediated by the autophagic network. Despite a slight mRNA increase, a significant decrease in protein expression supports the evidence that mTOR blockade impairs receptor recycling and directs EGFR to the degradative route. This observation is in line with the literature data demonstrating that mTOR activity is necessary for the recycling route of Transferrin receptors and plasma membrane lipids [41]. Indeed, mTORC1 inactivation reduces the expression of hepatocyte growth factor-regulated tyrosine kinase substrate (Hrs), a component of Endosomal Sorting Complex Required for Transport (ESCRT), which is crucial for lysosomal targeting of ubiquitylated cargoes [41]. This event leads to transferrin receptors and sphingomyelin delivery to lysosomes, independently of canonical autophagy. Even though Zhao et al. demonstrated that mTOR inhibition activates protein degradation by the ubiquitin-proteasome system as well as by autophagy [31], EGFR clearance does not involve proteasomal activity neither autophagy activation in our model.

In physiological conditions, EGFR phosphorylation at the plasma membrane leads to the recruitment of multiple effector proteins via recognition and binding of Src homology 2 (SH2) and phosphotyrosine-binding (PTB) domains to phosphotyrosine motifs on the receptor. Formation of the EGFR signalling complex, in turn, triggers a variety of signalling cascades involved in cell proliferation, migration, and survival. Interestingly, similar substrates are activated downstream of wt EGFR and EGFRΔIII, but with differing levels of intensity [13]. These pathways include the phosphoinositide 3 kinase (PI3K), mitogen activated protein kinase (MAPK), signal transducer and activator of transcription 3 (STAT3) pathways and the non-receptor tyrosine kinase Src [17]. In the tumoral masses, receptor alteration and impaired degradation are often associated to enhanced downstream oncogenic signalling [42,43]. As a consequence of EGFR endocytosis and degradation, we observed a clear and rapid inhibition of ERK1/2 kinases activity in our models. Intriguingly, it has been reported that mTOR inhibition increases ERK activity in some cancer models, and this event is likely due to the existence of a negative feedback loop between the PI3K/mTOR axis and MAPK/ERK pathway. In this context, other findings report that the mTOR inhibitor Rapamycin activates MAPK in a GBM cell line [44]. Despite these observations, other studies and early clinical trials highlighted that PI3K/mTOR inhibitors suppress the MEK/ERK pathway in other cancer types [45,46]. Noteworthily, PI3K inhibition led to ERK pathway suppression in HER2 amplified breast cancer [47].

ERK1/2 inhibition suggests a restriction of the proliferative potential of GBM cells. In fact, we observed that mTOR inhibition heavily affected cell proliferation, and potentiated the anti-proliferative/toxic effects of the drug TMZ. Based on this result and on our previous data [23,24], the effects of mTOR inhibition seem to converge to counter-act the in vitro glioma proliferation, migration and invasion.

We also found that mTOR inhibition elicits EGFR endocytosis by enhancing Src activation, as the administration of the Src inhibitor PP2 impairs the receptor internalisation when mTOR activity is suppressed. PP2 was also able to prevent the anti-proliferative activity of Torin1, thus suggesting that EGFR internalisation and Src activity are crucial for Torin1 efficacity. Src kinase is thought to be a crucial partner for EGFR in mediating oncogenesis, as they co-localize in lipid rafts and synergize to increase the mitogenic activity of EGFRs [48,49]. It has also been demonstrated that Src activity is required for clathrin phosphorylation and EGFR endocytosis upon EGF stimulation [32]. Our results show that Src activity is negatively regulated by mTOR kinase in GBM cells and indicate a crucial role for mTOR/Src axis on EGFR trafficking and localisation. We cannot exclude that the effects of mTOR inhibition on SRC and ERK activity occur via separate pathways; anyway, ERK inactivation could be the result of SRC-mediated EGFR delocalisation, positioning all the actors in one integrated route.

## 4. Materials and Methods

### 4.1. Cell Culture and Treatments

The human glioblastoma U87MG cell line was kindly provided by Prof. G. Velasco (Complutense University, Madrid, Spain). Human glioblastoma GL15 cells were kindly provided by Dr. E. Castigli, University of Perugia, Italy. Glioblastoma patient-derived culture GH2 were obtained from human GBM tumour samples from the Spanish National Cancer Centre (CNIO, Madrid, Spain) biobank (GH2). Histopathological typing was done according to the WHO criteria resulting as grade IV. All procedures involving samples of human origin were performed with the approval of the corresponding ethical committees from each institution as well as of the ethical committee of Complutense University (Madrid, Spain). Briefly, primary cultures were obtained by using the following procedure: tumour samples were mechanically and enzymatically dissociated with 0.12 mg/mL of collagenase type Ia from Clostridium histolyticum (Merck KGaA, Darmstadt, Germany) for 2 h at 37 °C and filtered using a 100 μm nylon filter (Millipore, Burlington, MA, USA). Cells were then plated and maintained as non-adherent neurospheres for at least 3 consecutive passages (with the aim of enriching the cultures in cells with stem-like properties) in a DMEM:Ham’s F-12 media (Lonza, Basel, Switzerland) supplemented with 1% penicillin–streptomycin (Lonza), 5 mM 4-(2-hydroxyethyl)-1-piperazineethanesulfonic acid (HEPES) buffer (Lonza), 2 mM ultraglutamine (Lonza), 20 ng/mL Epidermal growth factor (EGF) and Basic fibroblast growth factor (FGFb) (Gibco, Carlsbad, CA, USA), 2 μg/mL heparin sodium salt (Merck KGaA, Darmstadt, Germany), 1% B27 (Invitrogen, Carlsbad, CA, USA) and 1 µg/mL leukemia inhibitory factor (LIF, Millipore).

Human U87MG, GL15 were cultured in DMEM (Lonza, Basel, Switzerland), supplemented with 10% heat-inactivated Fetal Bovine Serum (FBS) (Euroclone, Milan, Italy) and 1% penicillin/streptomycin solution (Euroclone, Milan, Italy). Cells were grown at 37 °C in a 5% CO2 humidified atmosphere. shBECLIN1 and shCTR cells were prepared by lentiviral infection as previously described [23]. U87MG were infected by incubation with the supernatant of GFP-LC3-expressing retroviruses for 8h in the presence of 4 μg/mL poly-brene [50].

For autophagy induction and mTOR inhibition, cells were cultured in Earle’s Balanced Salt Solution (Merck KGaA, Darmstadt, Germany) or in the presence of 250 nM Torin 1 (Merck KGaA, Darmstadt, Germany), or 100 nM AZD8055 (Merck KGaA, Darmstadt, Germany). For SRC kinase inhibition, 20 µM PP2 (Merck KGaA, Darmstadt, Germany) was added to the culture medium. For endocytosis inhibition 100 μM Dynasore (Merck KGaA, Darmstadt, Germany) was added. To test endocyitic flux, 0.5 mg/mL Rhodamine B isothiocyanate Dextran (average MW 70 kDa) (Merck KGaA, Darmstadt, Germany) was employed. Amounts of 20 µM CQ and 3 µM MG132 were added to inhibit the late steps of autophagy and proteasome, respectively. For proliferation and apoptosis assays, 500 μM Temozolomide (Merck KGaA, Darmstadt, Germany) was employed.

### 4.2. Cell Lysis and Western Blotting

Protein extracts were prepared by lysing cells with the appropriate amount of Radioimmunoprecipitation assay (RIPA) Buffer (50 Mm Tris HCl pH 7.4, Triton 1%, Na Deoxycholate 0.25%, SDS 0.1%, 150 mM NaCl, 1 mM ethylenediaminetetraacetic acid (EDTA), 5 mM MgCl 2 supplemented with a protease inhibitors cocktail). After incubation on ice for 30 min, samples were centrifuged at 16,000× *g* for 30 min. Supernatants were recovered and protein concentrations were determined using a Lowry protein assay (Bio-Rad, Hercules, CA, USA). Proteins were separated by means SDS-PAGE and then electroblotted onto nitrocellulose (GE Healthcare, Life Sciences, Little Chalfont, Buckinghamshire, UK). After blocking, membranes were incubated with primary antibodies diluted in Phosphate buffered saline (PBS)/5% non-fat dry milk/0.1% Tween-20 overnight at 4 °C. Detection was obtained by using horseradish peroxidase-conjugated secondary antibody (Bio-Rad Laboratories, Milan, Italy) and visualized with enhanced chemiluminescence (ECL) plus (GE Healthcare, Life Sciences, Little Chalfont, Buckinghamshire, UK). The following primary antibodies were used: anti-P-p70SK, anti- p70SK, anti-P-mTOR, anti-mTOR, anti-LC3B, anti-P-ERK1/2 (Thr 202/Tyr 204), anti-ERK1/2, anti-Src, anti-P-Src (Y416) (Cell Signaling, Danvers, MA, USA), anti-p62, anti-BECLIN1, anti-HSP90 (Santa Cruz Biotechnology, Santa Cruz, CA, USA), anti-EGFR cat. #05-104 e #06-847 (Merck KGaA, Darmstadt, Germany), anti-β-Actin (Merck KGaA, Darmstadt, Germany), anti-β-catenin (BD Biosciences, Franklin Lakes, NJ, USA) and anti-Ubiquitin (EnzoLife Sciences, Villeurbanne, France).

### 4.3. Immunocytochemistry and Confocal Analysis

Cells were grown on coverslips and fixed with 4% PFA in PBS, followed by permeabilization with 0.1% Triton X-100 in PBS. EGFR (Upstate Biotechnology, USA), Calnexin, Tomm-20, Cathepsin-D (Santa Cruz Biotechnology, Santa Cruz, CA, USA), and Giantin (Abcam, Cambridge, UK) primary antibodies were incubated overnight at 4 °C and visualized by means of Alexa Fluor secondary antibodies (Invitrogen, Carlsbad, CA, USA).

After nuclear staining with Hoechst 33342 (Invitrogen, Carlsbad, CA, USA), coverslips were mounted in antifade (SlowFade; Invitrogen, Carlsbad, CA, USA) and examined under a confocal microscope (TCS SP8; Leica, Wetzlar, Germany), equipped with a 40 × 1.40–0.60 NA HCX Plan Apo oil BL objective at RT. Co-localisation analysis was performed by means of ImageJ software. The pixels of two 8-bit images (red and green channels of each image) are considered colocalized if their intensities are higher than the threshold of their channels (set at 50) and if the ratio of their intensity is higher than the ratio setting value (set at 50%).

Co-localisation was assessed by calculating the Pearson’s correlation coefficients of at least 10 cells analysed for each replicate. The Pearson’s correlation coefficient was expressed as mean ± SD. Three independent replicates were performed for each experiment.

To analyse the endocytic flux, Rhodamine B isothiocyanate Dextran was used, as previously described [51], and pictures were taken at the indicated time points on the confocal microscope. Rhodamine fluorescence intensity was quantified by ImageJ software.

### 4.4. Reverse Transcription and qPCR

500 ng of the total RNA isolated by the Direct-zol RNA Miniprep plus kit (Zymo Research, Irvine, CA, USA) and was retrotranscribed with SuperScript™ II Reverse Transcriptase (Bioline, GC biotech, Waddinxveen, The Netherlands), according to standard procedures. One µL of cDNA was employed to quantify the transcripts by real time RT-PCR using the SYBR Green Supermix (SensiFast SYBR Hi-ROX, Bioline, GC biotech, Waddinxveen, The Netherlands) and gene-specific primers. Real-time RT-PCR was performed by using a StepOne Real-Time PCR System (Applied Biosystems, Foster City, CA, USA). Relative quantity (RQ) was calculated normalizing for GAPDH gene and using a CTR sample as calibrator. Mean values and standard errors of RQ were generated from three biological replicates. The following primer sequences were used: EGFR-FW: 5′-AGGAAGAAGCTTGCTGGTAGC-3′; EGFR-REV: 5′-CTCTGGAAGACTTGTGGCTTG-3′; GAPDH-FW: 5′-CACCATCTTCCAGGAGCGAG-3′; REV 5′-CCTTCTCCATGGTGGTGAAGAC-3′.

### 4.5. Proliferation and Apoptosis Assays

GBM cells were grown on 24-well plates in the presence or absence of stimuli. Cell proliferation was assessed by counting cells at the indicated times in a Newbauer chamber, in the presence of Trypan blu, after trypsinization. Four counts for each condition were performed in each experiment. For apoptosis, detection cells were grown on coverslips and fixed with 4% PFA in PBS, followed by Hoechst 33342 staining. Fragmented and condensed nuclei (apoptotic) and intact nuclei were counted under a confocal microscope. At least 10 fields, at 20× magnification, were counted.

### 4.6. Statistical Analysis

All experiments were performed at least three times. GraphPad Prism and Excel software for windows were used for statistical analysis. Statistical significance was determined by using the Student’s *t*-test. A *p* value ≤ 0.05 was considered significant. Proliferation was analysed by using two- way ANOVA and apoptosis by using one-way ANOVA.

## 5. Conclusions

In our previous works, we demonstrated that nutrient starvation and mTOR inhibition strongly impaired the EMT process and Wnt/β-catenin signalling in GBM cells, through β-catenin colocalisation with N-cadherin in sub-membrane areas [23,24,25]. Here, we provide the first evidence for an involvement of the mTOR/PI3K pathway in RTK trafficking and expression, thus enforcing the proof of concept that mTOR complexes may be further considered as potential tools for pharmacological interventions in cancer, including GBM [36,52].

Currently, EGFR is a promising candidate for therapeutic approaches against cancer. Notably, its alterations show a homogenous and tissue-specific distribution across the tumour mass, and are scarcely detectable in the healthy brain and in other tissues, making it an attractive target for innovative therapeutic intervention. In this context, the clinical benefits of EGFR inhibitors have been observed in different types of tumour. For instance, both monoclonal antibodies and EGFR-directed inhibitors are employed and continuously optimized as antitumorals. However, the occurrence of a multitude of adaptive mechanisms often leads to the appearance of drug resistance [53,54]. Moreover, the effectiveness of EGFR-based therapies has not yet been validated in GBM. The observations here reported may help to set the bases for design novel therapeutic strategies to fight GBM, based on mTOR pharmacological inhibition.

## Supplementary Materials

The following are available online at https://www.mdpi.com/2072-6694/12/8/2266/s1, Suppl. Method, Figure S1: EGFR internalises into GBM cells upon mTOR inhibition, Figure S2: EGFR is delivered to lysosomes upon mTOR inhibition in GL15 and in primary GH2 GBM cells, Figure S3: mTOR inhibition leads to EGFR degradation in GL15 and GH2 cells.

## References

[1] Lemmon M.A., Schlessinger J., Ferguson K.M. (2014). The EGFR family: Not so prototypical receptor tyrosine kinases. Cold Spring Harb. Perspect. Biol..

[2] Ceresa B.P., Peterson J.L. (2014). Cell and Molecular Biology of Epidermal Growth Factor Receptor. Int. Rev. Cell Mol. Biol..

[3] Wee P., Wang Z. (2017). Epidermal growth factor receptor cell proliferation signaling pathways. Cancers.

[4] Tomas A., Futter C.E., Eden E.R. (2014). EGF receptor trafficking: Consequences for signaling and cancer. Trends Cell Biol..

[5] Sigismund S., Woelk T., Puri C., Maspero E., Tacchetti C., Transidico P., Di Fiore P.P., Polo S. (2005). Clathrin-independent endocytosis of ubiquitinated cargos. Proc. Natl. Acad. Sci. USA.

[6] Critchley W., Pellet-Many C., Ringham-Terry B., Harrison M., Zachary I., Ponnambalam S. (2018). Receptor Tyrosine Kinase Ubiquitination and De-Ubiquitination in Signal Transduction and Receptor Trafficking. Cell.

[7] Goh L.K., Sorkin A. (2013). Endocytosis of receptor tyrosine kinases. Cold Spring Harb. Perspect. Biol..

[8] Caldieri G., Barbieri E., Nappo G., Raimondi A., Bonora M., Conte A., Verhoef L.G., Confalonieri S., Malabarba M.G., Bianchi F. (2017). Reticulon3-dependent ER-PM contact sites control EGFR non-clathrin endocytosis. Science.

[9] Bakker J., Spits M., Neefjes J., Berlin I. (2017). The EGFR odyssey—From activation to destruction in space and time. J. Cell Sci..

[10] Sigismund S., Avanzato D., Lanzetti L. (2018). Emerging functions of the EGFR in cancer. Mol. Oncol..

[11] Roskoski R. (2014). The ErbB/HER family of protein-tyrosine kinases and cancer. Pharmacol. Res..

[12] Saadeh F.S., Mahfouz R., Assi H.I. (2018). Egfr as a clinical marker in glioblastomas and other gliomas. Int. J. Biol. Markers.

[13] An Z., Aksoy O., Zheng T., Fan Q.W., Weiss W.A. (2018). Epidermal growth factor receptor and EGFRvIII in glioblastoma: Signaling pathways and targeted therapies. Oncogene.

[14] Xu H., Zong H., Ma C., Ming X., Shang M., Li K., He X., Du H., Cao L. (2017). Epidermal growth factor receptor in glioblastoma. Oncol. Lett..

[15] Mizoguchi M., Betensky R.A., Batchelor T.T., Bernay D.C., Louis D.N., Nutt C.L. (2006). Activation of STAT3, MAPK, and AKT in Malignant Astrocytic Gliomas. J. Neuropathol. Exp. Neurol..

[16] Grandal M.V., Zandi R., Pedersen M.W., Willumsen B.M., van Deurs B., Poulsen H.S. (2007). EGFRvIII escapes down-regulation due to impaired internalization and sorting to lysosomes. Carcinogenesis.

[17] Huang P.H., Xu A.M., White F.M. (2009). Oncogenic EGFR signaling networks in glioma. Sci. Signal..

[18] Wilson K.J., Gilmore J.L., Foley J., Lemmon M.A., Riese D.J. (2009). Functional selectivity of EGF family peptide growth factors: Implications for cancer. Pharmacol. Ther..

[19] Wilson K.J., Mill C., Lambert S., Buchman J., Wilson T.R., Hernandez-Gordillo V., Gallo R.M., Ades L.M.C., Settleman J., Riese D.J. (2012). EGFR ligands exhibit functional differences in models of paracrine and autocrine signaling. Growth Factors.

[20] Saxton R.A., Sabatini D. (2017). mTOR Signaling in Growth, Metabolism and Disease. Cell.

[21] Zheng Y., Jiang Y. (2017). mTOR Inhibitors at a Glance. Physiol. Behav..

[22] Mizushima N. (2018). A brief history of autophagy from cell biology to physiology and disease. Nat. Cell Biol..

[23] Catalano M., D’Alessandro G., Lepore F., Corazzari M., Caldarola S., Valacca C., Faienza F., Esposito V., Limatola C., Cecconi F. (2015). Autophagy induction impairs migration and invasion by reversing EMT in glioblastoma cells. Mol. Oncol..

[24] Colella B., Faienza F., Carinci M., Alessandro G.D., Catalano M., Santoro A., Cecconi F., Limatola C., Di Bartolomeo S. (2019). Autophagy induction impairs Wnt/β-catenin signalling through β-catenin relocalisation in glioblastoma cells. Cell. Signal..

[25] Colella B., Faienza F., Di Bartolomeo S. (2019). EMT regulation by autophagy: A new perspective in glioblastoma biology. Cancers.

[26] Fraser J., Cabodevilla A.G., Simpson J., Gammoh N. (2017). Interplay of autophagy, receptor tyrosine kinase signalling and endocytic trafficking. Essays Biochem..

[27] Bell E.S., Coelho P.P., Ratcliffe C.D.H., Rajadurai C.V., Peschard P., Vaillancourt R., Zuo D., Park M. (2019). LC3C-Mediated Autophagy Selectively Regulates the Met RTK and HGF-Stimulated Migration and Invasion. Cell Rep..

[28] Larrue C., Saland E., Boutzen H., Vergez F., David M., Joffre C., Hospital M.A., Tamburini J., Delabesse E., Manenti S. (2016). Proteasome inhibitors induce FLT3-ITD degradation through autophagy in AML cells. Blood.

[29] Tan X., Thapa N., Sun Y., Anderson R.A. (2015). A kinase-independent role for EGF receptor in autophagy initiation. Cell.

[30] Klionsky D.J., Abdelmohsen K., Abe A., Abedin M.J., Abeliovich H., Arozena A.A., Adachi H., Adams C.M., Adams P.D., Adeli K. (2016). Guidelines for the use and interpretation of assays for monitoring autophagy (3rd edition). Autophagy.

[31] Zhao J., Zhai B., Gygi S.P., Goldberg A.L. (2015). MTOR inhibition activates overall protein degradation by the ubiquitin proteasome system as well as by autophagy. Proc. Natl. Acad. Sci. USA.

[32] Wilde A., Beattie E.C., Lem L., Riethof D.A., Liu S.H., Mobley W.C., Soriano P., Brodsky F.M. (1999). EGF receptor signaling stimulates SRC kinase phosphorylation of clathrin, influencing clathrin redistribution and EGF uptake. Cell.

[33] Ma Y.C., Huang X.Y. (2002). Novel regulation and function of Src tyrosine kinase. Cell. Mol. Life Sci..

[34] Thoreen C.C., Kang S.A., Chang J.W., Liu Q., Zhang J., Gao Y., Reichling L.J., Sim T., Sabatini D.M., Gray N.S. (2009). An ATP-competitive mammalian target of rapamycin inhibitor reveals rapamycin-resistant functions of mTORC1. J. Biol. Chem..

[35] Dowling R.J.O., Topisirovic I., Alain T., Bidinosti M., Bruno D., Petroulakis E., Wang X., Larsson O., Selvaraj A., Kozma S.C. (2010). mTORC1-mediated cell proliferation, but not cell growth, controlled by the 4E-BPs. Science.

[36] Prasad G., Sottero T., Yang X., Mueller S., James C.D., Weiss W.A., Polley M., Ozawa T., Berger M.S., Aftab D.T. (2011). Glioblastoma and Implications for Combination Therapy With Temozolomide. Neuro. Oncol..

[37] Sondka Z., Bamford S., Cole C.G., Ward S.A., Dunham I., Forbes S.A. (2018). The COSMIC Cancer Gene Census: Describing genetic dysfunction across all human cancers. Nat. Rev. Cancer.

[38] Shan Y., Eastwood M.P., Zhang X., Kim E.T., Arkhipov A., Dror R.O., Jumper J., Kuriyan J., Shaw D.E. (2012). Oncogenic mutations counteract intrinsic disorder in the EGFR kinase and promote receptor dimerization. Cell.

[39] Wang Y.N., Yamaguchi H., Hsu J.M., Hung M.C. (2010). Nuclear trafficking of the epidermal growth factor receptor family membrane proteins. Oncogene.

[40] Demory M.L., Boerner J.L., Davidson R., Faust W., Miyake T., Lee I., Hüttemann M., Douglas R., Haddad G., Parsons S.J. (2009). Epidermal growth factor receptor translocation to the mitochondria. J. Biol. Chem..

[41] Dauner K., Eid W., Raghupathy R., Presley J.F., Zha X. (2017). MTOR complex 1 activity is required to maintain the canonical endocytic recycling pathway against lysosomal delivery. J. Biol. Chem..

[42] Sorkin A., Von Zastrow M. (2009). Endocytosis and signalling: Intertwining molecular networks. Nat. Rev. Mol. Cell Biol..

[43] Roepstorff K., Grøvdal L., Grandal M., Lerdrup M., Van Deurs B. (2008). Endocytic downregulation of ErbB receptors: Mechanisms and relevance in cancer. Histochem. Cell Biol..

[44] Albert L., Karsy M., Murali R., Jhanwar-Uniyal M. (2009). Inhibition of mTOR activates the MAPK pathway in glioblastoma multiforme. Cancer Genom. Proteom..

[45] Edelman G., Bedell C., Shapiro G., Pandya S.S., Kwak E.L., Scheffold C., Nguyen L.T., Laird A., Baselga J., Rodon J. (2010). A phase I dose-escalation study of XL147 (SAR245408), a PI3K inhibitor administered orally to patients (pts) with advanced malignancies. J. Clin. Oncol..

[46] King W.G., Mattaliano M.D., Chan T.O., Tsichlis P.N., Brugge J.S. (1997). Phosphatidylinositol 3-kinase is required for integrin-stimulated AKT and Raf-1/mitogen-activated protein kinase pathway activation. Mol. Cell. Biol..

[47] Ebi H., Costa C., Faber A.C., Nishtala M., Kotani H., Juric D., Della Pelle P., Song Y., Yano S., Mino-Kenudson M. (2013). PI3K regulates MEK/ERK signaling in breast cancer via the Rac-GEF, P-Rex1. Proc. Natl. Acad. Sci. USA.

[48] Donepudi M., Resh M.D. (2008). c-Src trafficking and co-localization with the EGF receptor promotes EGF ligand-independent EGF receptor activation and signaling. Cell. Signal..

[49] Irwin M.E., Bohin N., Boerner J.L. (2011). Src family kinases mediate epidermal growth factor receptor signaling from lipid rafts in breast cancer cells. Cancer Biol. Ther..

[50] Fimia G.M., Stoykova A., Romagnoli A., Giunta L., Di Bartolomeo S., Nardacci R., Corazzari M., Fuoco C., Ucar A., Schwartz P. (2007). Ambra1 regulates autophagy and development of the nervous system. Nature.

[51] Li L., Wan T., Wan M., Liu B., Cheng R., Zhang R. (2015). The effect of the size of fluorescent dextran on its endocytic pathway. Cell Biol. Int..

[52] Roskoski R. (2020). Properties of FDA-approved small molecule protein kinase inhibitors: A 2020 update. Pharmacol. Res..

[53] Westphal M., Maire C.L., Lamszus K. (2017). EGFR as a Target for Glioblastoma Treatment: An Unfulfilled Promise. CNS Drugs.

[54] Artene S.A., Tuţă C., Dragoi A., Alexandru O., Stefana Oana P., Tache D.E., Dănciulescu M.M., Boldeanu M.V., Siloşi C.A., Dricu A. (2018). Current and emerging EGFR therapies for glioblastoma. J. Immunoass. Immunochem..

