# Supplementary material for: mTOR Inhibition Leads to Src-Mediated EGFR Internalisation and Degradation in Glioma Cells
Source: Cancers (Basel). 2020 Aug 13;12(8):2266. doi: 10.3390/cancers12082266 (PMC7464593; doi:10.3390/cancers12082266)

Supplementary Material

Supplementary method

Immunoprecipitation

EGFR immunoprecipitation was performed as described (Conte and Sigismund, Methods to investigate EGFR Ubiquitination, ErbB Recptor Signaling: Methods and Protocols, DOI 10.1007/978-1-4939-7219-7_5,). Briefly, U87MG cells, grown in absence or in presence of 250 nM Torin1 and pre-incubated with 3 µM MG132, were lysed with RIPA buffer containing 1% SDS (50mM Tris-HCl pH7.4, 150mM NaCl, 1mM EDTA, 1% Triton, 1% Na deoxycholate, 1% SDS plus proteases and phosphatases inhibitors) and let on ice 15 min before centrifugation at 110000g for 20 min. Supernatants were the diluted with RIPA buffer w/o SDS to reach a final concentration of 0.2% SDS. After protein quantification, 900 µg of cell lysates were incubated with 1 µg anti-EGFR #05-104 (Merck, Kenilworth, USA) or with mouse IgG over night at 4°C on a wheel shaker. Lysates were then incubated with 30 µl of Protein A/G-conjugated sepharose beads for 1 hour in the wheel shaker. After 4 rounds of centrifugation (400g, 5 min) and washes with RIPA buffer 0.2% SDS, beads were resuspended in 40 µl Laemlii buffer and boiled at 95°C for 5 min. 20 µl were loaded for western blotting analysis.

Supplementary figures

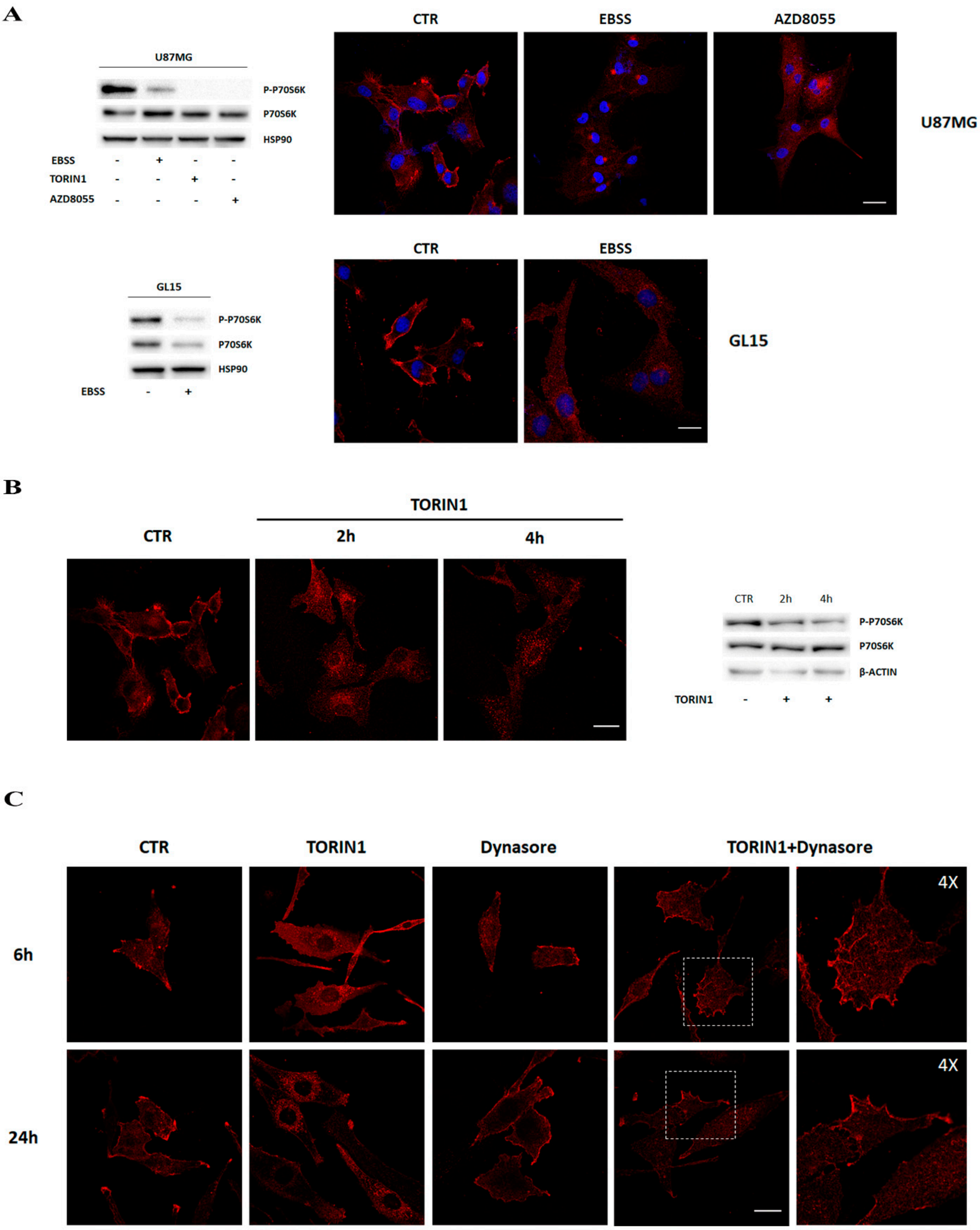

Supplementary Fig. S1 EGFR internalises into GBM cells upon mTOR inhibition. (A) U87MG (upper panels) and GL15 (lower panels) were cultured in complete DMEM (CTR) or aminoacid- and serum- free medium (EBSS) or in presence of 100nM AZD8055–for 18h. Immunocytochemistry and confocal analysis for EGFR localisation (red) were then performed. Hoechst 33342 was used to stain nuclei (blue). Scale bar, 30 µM. Western

blot analysis of P-p70S6K and p70S6K was performed to check mTOR pathway inhibition by Torin1. HSP90 was used as loading control. The blots are representative of three independent experiments. **(B)** Immunocytochemistry and confocal analysis for EGFR localisation (red) was performed in GL15 cells, upon 2h and 4h Torin1 treatment. Scale bar, 30 μM. Western blot analysis of P-p70S6K and p70S6K was performed to check mTOR pathway inhibition by Torin1. β-ACTIN was used as loading control. **(C)** Immunocytochemistry and confocal analysis for EGFR localisation (red) were performed in GL15 cells, upon 6h and 24h Torin1

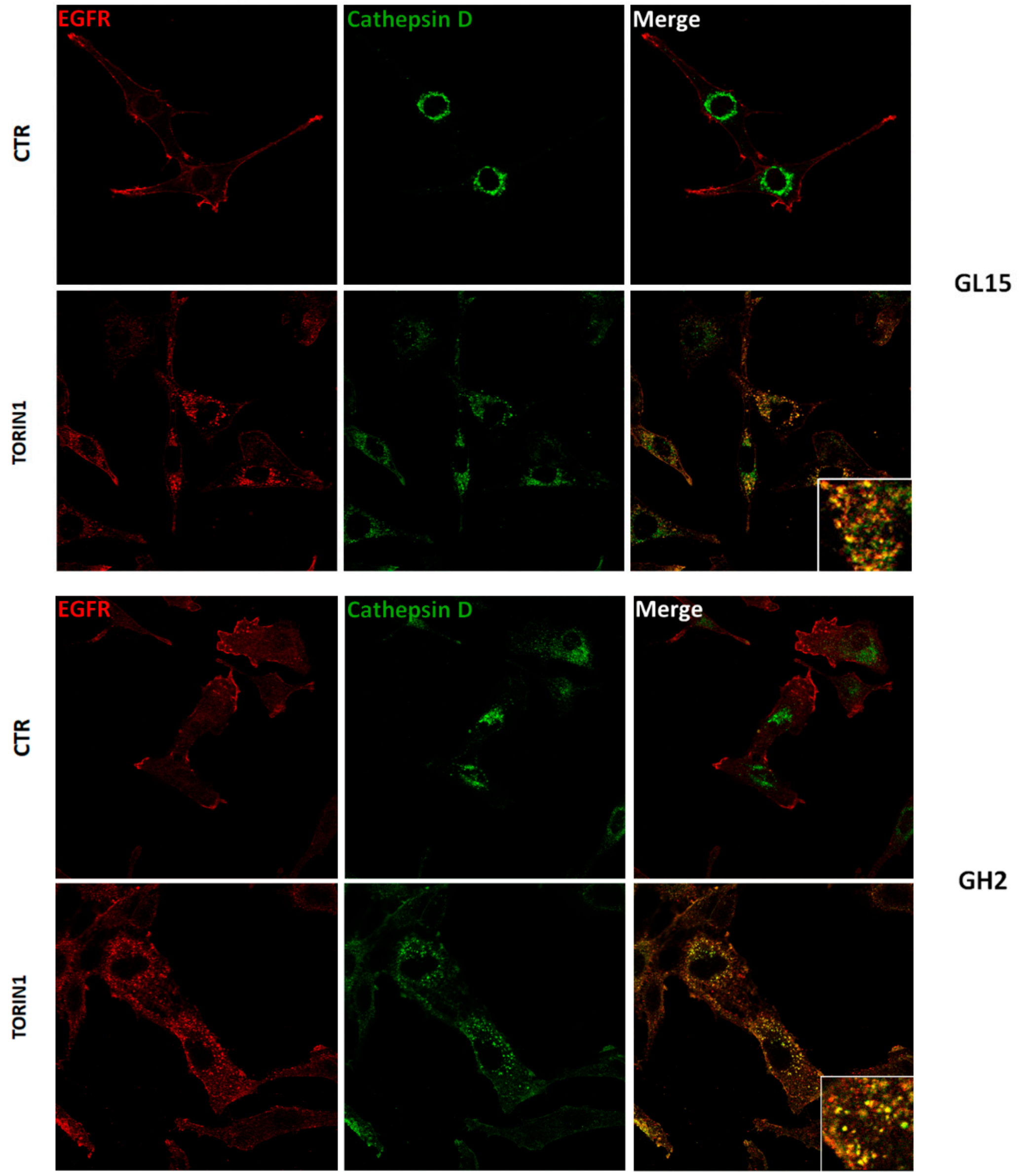

treatment in presence or not of 100 μM Dynasore. Scale bar, 30 μM. A 4X magnification is shown for the right panels representing cells treated with Torin1 plus Dynasore.

Supplementary Fig.S2 EGFR is delivered to lysosomes upon mTOR inhibition in GL15 and in primary GH2 GBM cells. GL15 and GH2 GBM cells, cultured in complete DMEM medium in presence (TORIN1) or not (CTR) of 250nM Torin1, were subjected to immunocytochemistry and confocal analysis for EGFR (red) and for lysosomes (green). The images showing the merge of the two signals are shown in the right panels. Inset containing higher magnification views of the merge images are also shown. Scale bar, 30 μM. Colocalization was assessed by calculating the Pearson's correlation coefficient r (for GL15: Ctr, 0.28 ± 0.01; Torin1, 0.7 ± 0.05. for GH2: Ctr, 0.2 ± 0.01; Torin1, 0.68 ± 0.02).

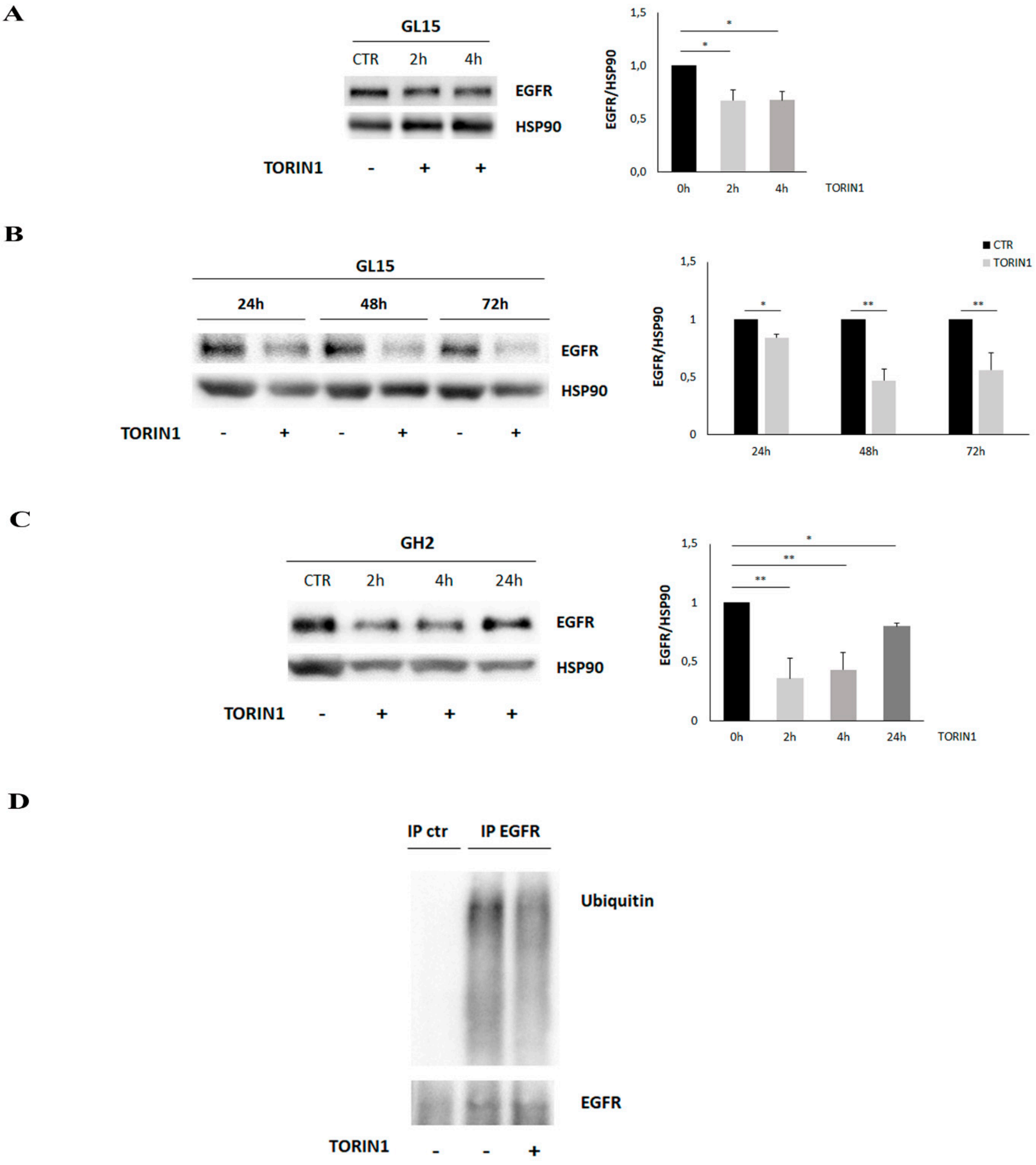

Supplementary Fig.S3 mTOR inhibition leads to EGFR degradation in GL15 and GH2 cells. (A) and **(B)** GL15 were cultured in complete DMEM medium in presence (TORIN1) or not (CTR) of 250nM Torin1 and analysed at the indicated time points. Western blot analyses were performed by using a specific antibody for EGFR. HSP90 was used as loading control. **(C)** GH2 cells were cultured in complete DMEM medium in presence (TORIN1) or not (CTR) of 250nM Torin1 and analysed at the indicated time points. **(D)** U87MG cells were stimulated (+) or not (-) with 250nM Torin1 for 24 hours in presence of 3 μM MG132 and immunoprecipitation analysis was performed by using anti-EGFR (IP EGFR) antibody and mouse IgG as control (IP ctr). Western blot analysis was performed by using anti-EGFR and anti-Ubiquitin (Ub). The graphs represent the mean ± SE of three different experiments. Statistical significance: * $P < .05$ Student t-test ** $P < .01$ Student t-test.

Whole blots

Related to Fig.1A

anti-phospho-P70S6K

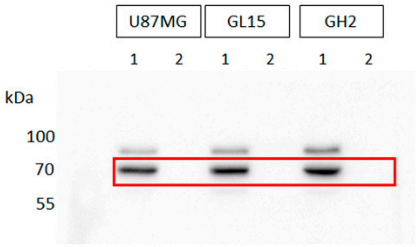

100 : 5 : 100 : 6 : 100 : 5

anti-P70S6K

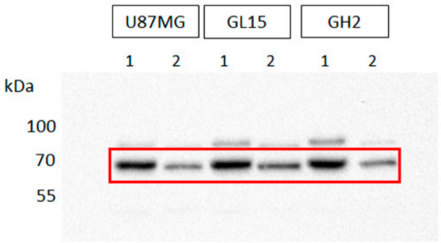

100 : 42 : 100 : 60 : 100 : 40

anti-β-Actin

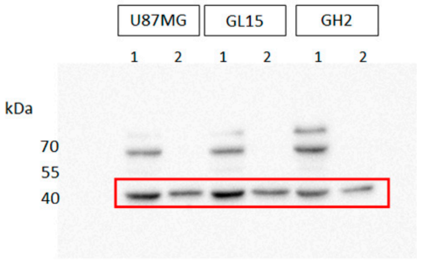

100 : 68 : 100 : 62 : 100 : 62

Related to Fig.2A

anti-Beclin1

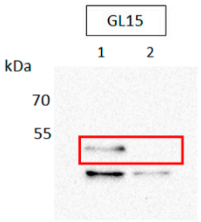

100 : 18

anti-P62

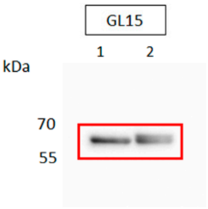

100 : 82

anti-LC3

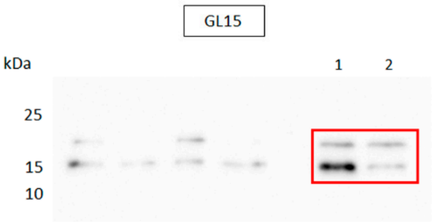

100 : 28

anti-β-Actin

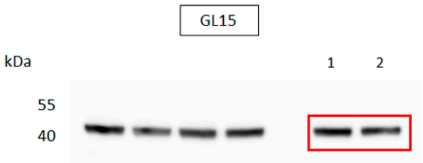

100 : 81

Related to Fig.4A

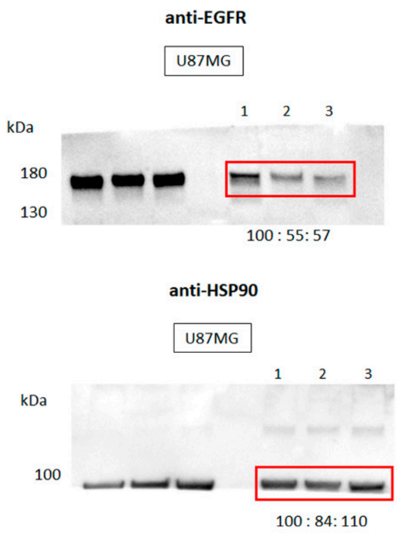

Related to Fig.4B

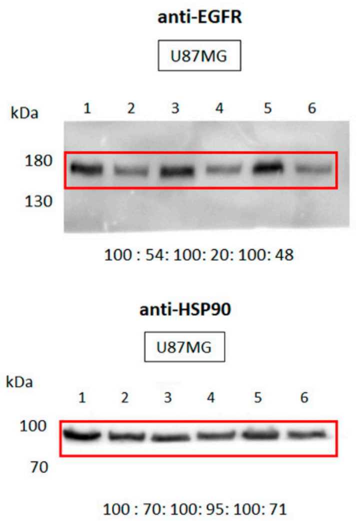

Related to Fig.4D

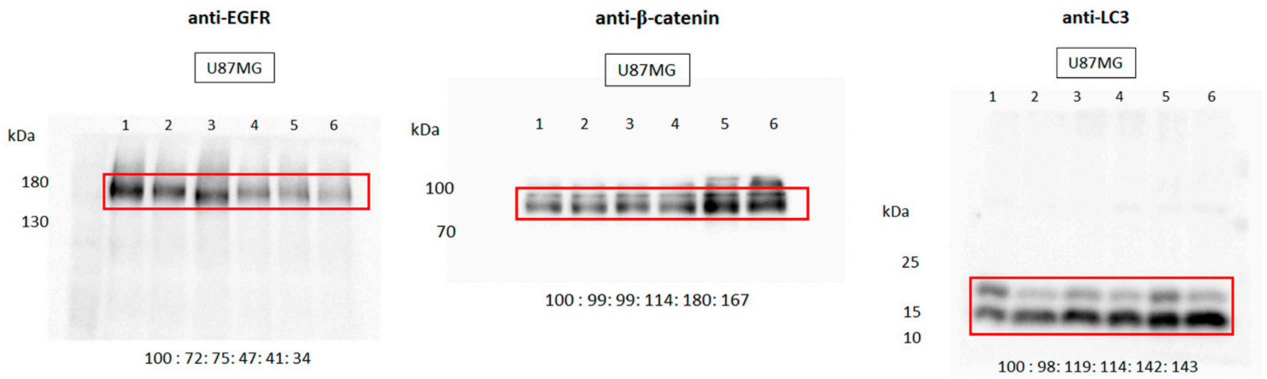

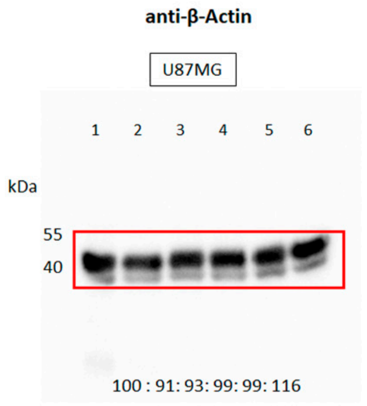

Related to Fig.4E

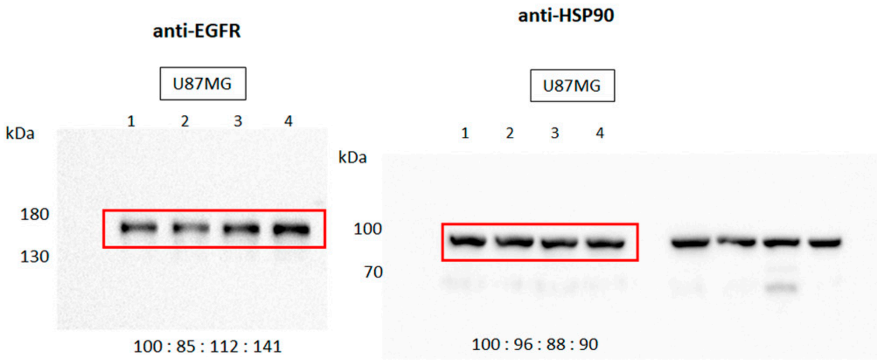

Related to Fig.5A

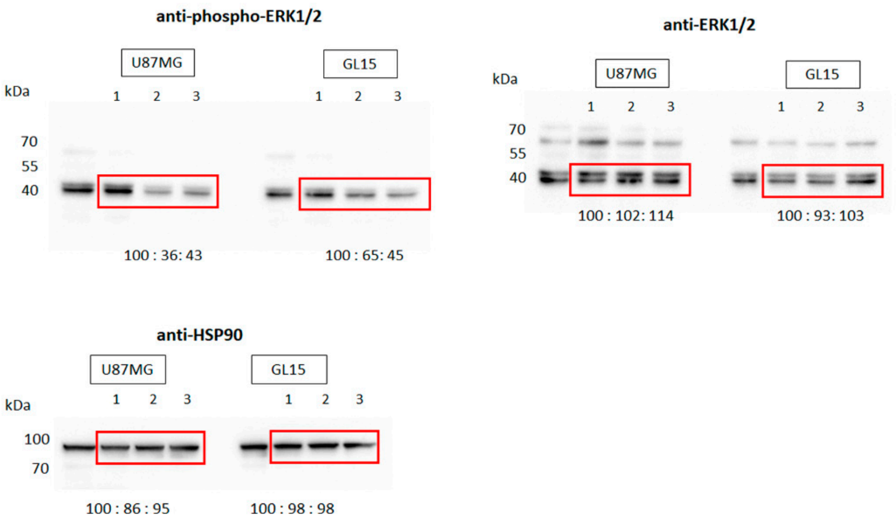

Related to Fig.5B

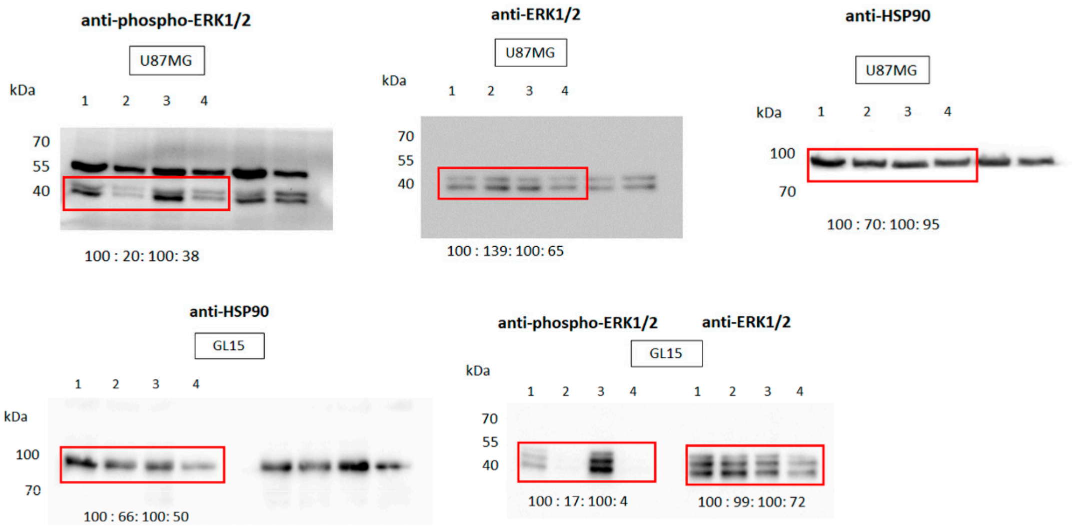

Related to Fig.5C

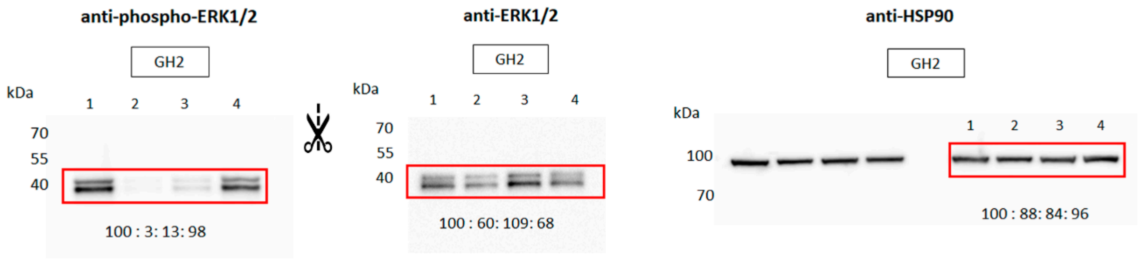

Related to Fig.6A

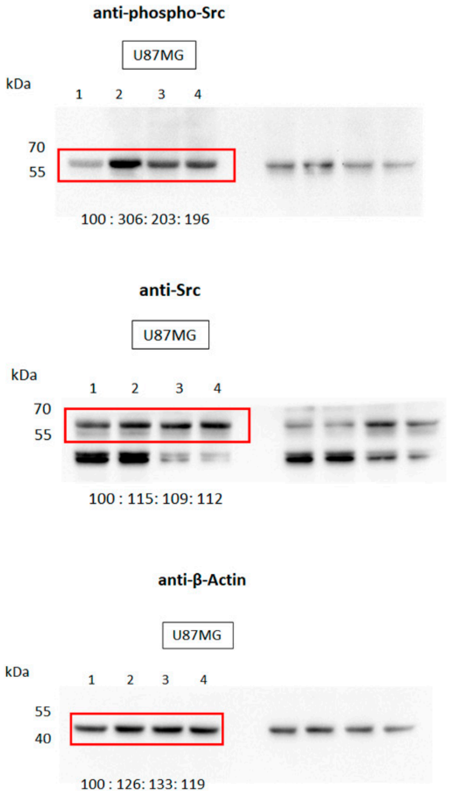

Related to Fig.6B

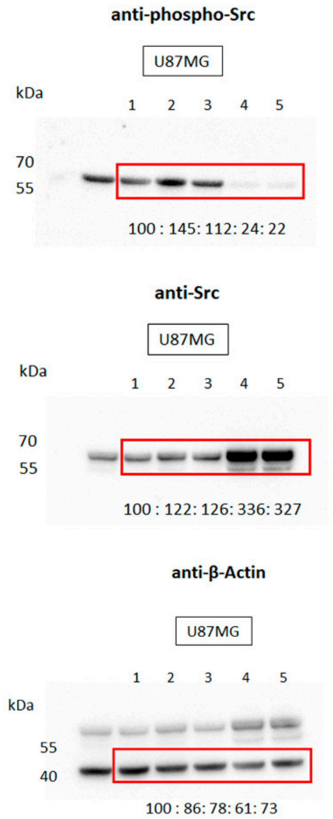

Related to Fig.6D

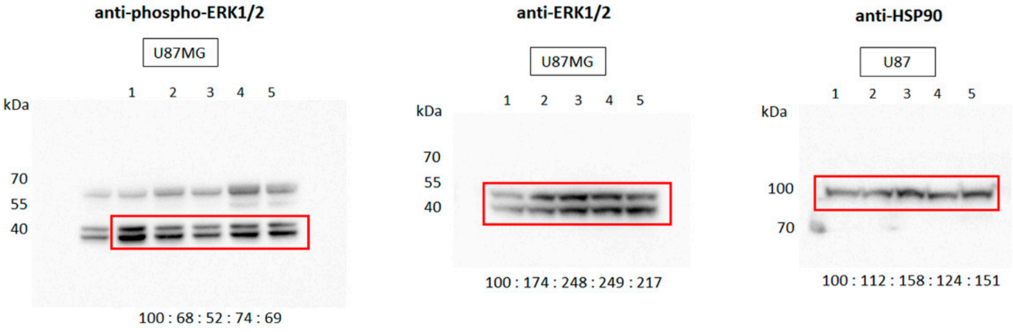

Related to Supplementary Fig.S3 A

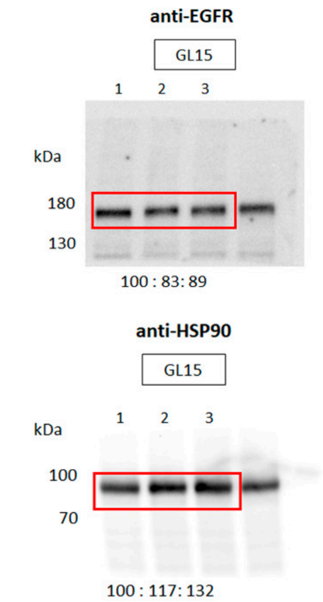

Related to Supplementary Fig.S3 C

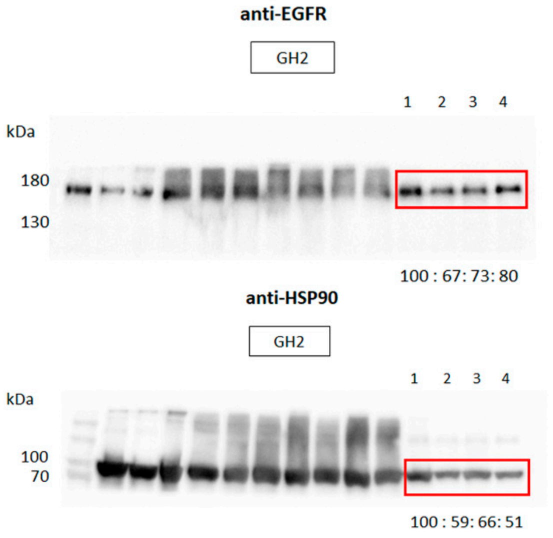

Related to Supplementary Fig.S3 B

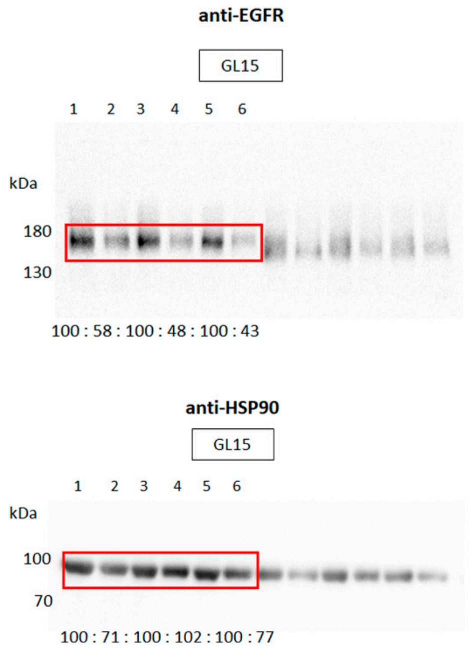

Related to Supplementary Fig.S3 D

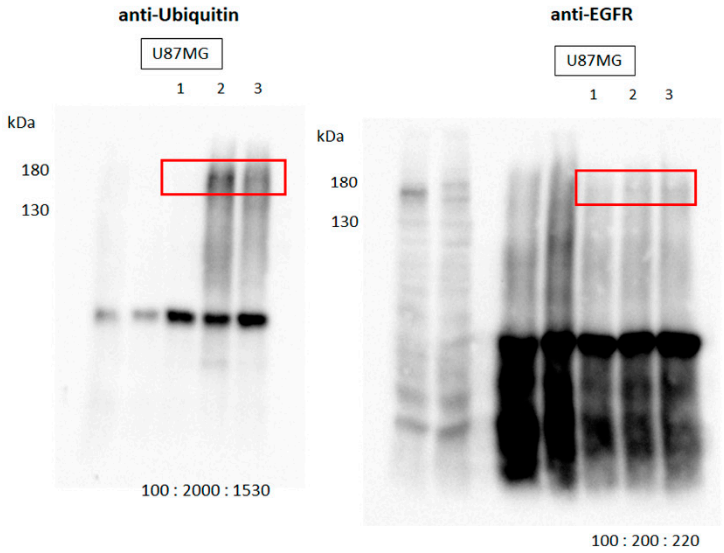

Supplement: Supplementary file 1 [file cancers-12-02266-s001.pdf]
